# Accurate positioning for head and neck cancer patients using 2D and 3D image guidance

**DOI:** 10.1120/jacmp.v12i1.3270

**Published:** 2010-10-27

**Authors:** Hyejoo Kang, Dale M. Lovelock, Ellen D. Yorke, Sergey Kriminiski, Nancy Lee, Howard I. Amols

**Affiliations:** ^1^ Department of Medical Physics, and Department of Radiation Oncology New York NY USA; ^2^ Department of Radiation Oncology Memorial Sloan Kettering Cancer Center New York NY USA

**Keywords:** head and neck, patient positioning, image guidance, CBCT

## Abstract

Our goal is to determine an optimized image‐guided setup by comparing setup errors determined by two‐dimensional (2D) and three‐dimensional (3D) image guidance for head and neck cancer (HNC) patients immobilized by customized thermoplastic masks. Nine patients received weekly imaging sessions, for a total of 54, throughout treatment. Patients were first set up by matching lasers to surface marks (initial) and then translationally corrected using manual registration of orthogonal kilovoltage (kV) radiographs with DRRs (2D‐2D) on bony anatomy. A kV cone beam CT (kVCBCT) was acquired and manually registered to the simulation CT using only translations (3D‐3D) on the same bony anatomy to determine further translational corrections. After treatment, a second set of kVCBCT was acquired to assess intrafractional motion. Averaged over all sessions, 2D‐2D registration led to translational corrections from initial setup of 3.5±2.2 (range 0–8) mm. The addition of 3D‐3D registration resulted in only small incremental adjustment (0.8±1.5mm). We retrospectively calculated patient setup rotation errors using an automatic rigid‐body algorithm with 6 degrees of freedom (DoF) on regions of interest (ROI) of in‐field bony anatomy (mainly the C2 vertebral body). Small rotations were determined for most of the imaging sessions; however, occasionally rotations >3° were observed. The calculated intrafractional motion with automatic registration was <3.5 mm for eight patients, and <2° for all patients. We conclude that daily manual 2D‐2D registration on radiographs reduces positioning errors for mask‐immobilized HNC patients in most cases, and is easily implemented. 3D‐3D registration adds little improvement over 2D‐2D registration without correcting rotational errors. We also conclude that thermoplastic masks are effective for patient immobilization.

PACS number: 87.53.Kn

## I. INTRODUCTION

Accurate positioning in the radiation treatment of head and neck cancer (HNC) patients is important since multiple critical organs surround the target. Setup errors can result in significant underdose to the tumor and/or overdose to one or more critical organs. HNC patients are often immobilized in a fixed position with custom‐made thermoplastic masks. To ensure accuracy on the treatment couch, patient positioning adjustments are made by aligning surface markers (i.e, tattoos), placed at the time of simulation. For the purpose of this paper, we will refer this as the “initial” setup process. In common clinical practice, after the initial setup, corrective shifts are periodically (weekly) determined by two‐dimensional (2D) registration of an orthogonal radiograph pair acquired while patients are on the treatment couch with corresponding radiographs or digitally reconstructed radiographs (DRR) from simulation (2D‐2D registration). The corrective shifts are applied and are utilized for subsequent treatments until the next imaging is done, typically one week later. Studies of the accuracy of the standard setup method include registration of plane radiographs (2D‐2D),^(^ ^1^ ^–^ ^6^ ^)^ volumetric registrations on three‐dimensional (3D) images (3D‐3D),^(^ ^7^ ^–^ ^9^ ^)^ and 2D radiographs and volumetric registrations (2D‐3D).^(^ ^10^ ^–^ ^12^ ^)^ Studies based on 2D‐2D bony anatomy registration report translational errors of approximately 5 mm in any direction. The average translation setup error in three dimensions, measured with an infrared surface marker system, is reported to be up to 6.97 mm for daily patient setup with three‐point laser alignment.^(^ ^13^ ^–^ ^14^ ^)^ More advanced image‐guidance methods using 3D imaging at treatment have also been studied. These include corrections based on registration of the planning computerized tomography^(^ ^3^ ^)^ scan with in‐room CT^(^ ^15^ ^–^ ^16^ ^)^ or kV or MV cone beam CT.^(^ ^12^ ^,^ ^17^ ^–^ ^18^ ^)^

The advent of treatment machines equipped with both kV radiographic and cone beam imaging capability has resulted in a choice of setup procedures. In principle, 3D imaging methods are expected to better visualize internal landmarks and lead to more accurate setups than 2D imaging. 3D‐3D registrations are more capable of detecting rotational setup errors^(^ ^7^ ^–^ ^9^ ^,^ ^16^ ^,^ ^18^ ^)^ which, if large enough (above 3°) can result in more than 3 mm displacement of structures that are greater than 5 cm from isocenter.

However, the potential benefits of patient positioning techniques based on 3D over 2D imaging need to be addressed. 3D imaging increases a patient's radiation exposure, and requires a longer time and more sophisticated therapist skills to acquire and to register. Most of all, 3D techniques require more expensive facilities equipped with CBCT or in‐room CT. It is therefore useful to address the potential advantages of using 3D techniques and develop an image‐guided setup method, which can be easily and broadly implemented in the clinic and maximizes clinical benefit while minimizing time and clinical effort.

In our study, we investigated whether 3D‐3D registrations significantly reduce setup errors compared to 2D‐2D registration for HNC patients immobilized with a custom thermoplastic mask. We also measured intrafractional errors to assess the immobilization performance of a thermoplastic mask.

## II. MATERIALS AND METHODS

### A. Simulation and treatment setup

Nine patients with various HNC cancers consented to this study. The primary tumors were treated with IMRT delivered using 4 to 10 beams for 25 to 35 fractions. The primary tumor sites for the patients are listed in Table 1. Some patients were also treated for nodal disease in the lower neck with an AP field with a cord block.

**Table 1:** Primary tumor sites of the patients for our study.

*Patient*	*Disease Sites*
1	Oral Cavity
2	Larynx
3	Parotid Bed
4	Right Sinus
5	Thyroid
6	Thyroid
7	Nasal Cavity
8	Thyroid
9	Nasopharynx

For each patient, a thermoplastic face mask (Orfit Industries, Wijnegen, Belgium) was custom‐made at simulation: the patient's head was on a commercial, standard headrest with mask and headrest affixed to a baseplate. Special attention is required to yield reproducible immobilization as the patient must be prevented from moving inside the mask while it is hardening. CT scans with a slice thickness of 3 mm were acquired and a physician determined the treatment isocenter based on the CT scan. One to three alignment tattoos were placed on the chest to localize the isocenter on the neck, and isolines were drawn on the mask for alignment with the room laser at treatment. Seven patients were immobilized with 5‐point masks and two patients with 3‐point masks. The masks of the first five patients were cut open around the eyes.

During treatment, at weekly imaging session following the initial setup, an orthogonal pair of kV radiographs (usually AP and lateral) was acquired using the Varian On Board Imaging system (OBI) (Varian Medical Systems, Palo Alto, CA), and manually registered to the simulation DRRs by the therapists using the Varian OBI application to determine setup errors and the necessary corrective couch shifts (manual 2D‐2D). This part of the setup is our departmental standard for conventionally fractionated HNC patients on machines with kV on‐board imaging. For the patients in this study, after these shifts, a kVCBCT was acquired and manually registered by the therapists with the simulation CT using the Varian OBI application (manual 3D‐3D) to determine and further correct the setup errors. Finally, the treatment position for that day was confirmed by acquiring and registering a second kV orthogonal pair with the DRRs. Occasionally (3 out of 54 imaging days), the patients were moved further because setup errors were observed on the second pair of radiographs and then a third kV orthogonal pair was acquired for the final confirmation. There were 54 image‐guided treatment sessions, 5 to 7 sessions for each patient. Following the protocol for this study, the same shifts were applied until the next imaging session, one week later.

The manual 2D‐2D registration was based upon the therapist's visual evaluation of the coincidence of bony anatomy (cervical vertebrae) in the superimposed kV radiographs and the DRRs. Shifts above 1 mm in any direction were applied through the console computer. If a large rotation was observed in the first set of kV radiographs, the mask was removed and the patient was repositioned. The initial setup was then repeated.

Since the bony anatomy in the HNC regions is deformable, the results of any rigid‐body registration depend on the location of the selected region of interest.^(^ ^19^ ^–^ ^20^ ^)^ Overall agreement of the cervical spine positions on the two images was the main registration criterion for the manual registration. In particular, the C2 vertebral body was considered an important ROI for all patients except for one whose target was distant from C2 (the right sinus). At our and other institutions,^(^ ^6^ ^,^ ^19^ ^)^ selecting C2 as a ROI for registration is accepted for targets that are close to the cervical vertebrae because the isocenters are often close to C1 to C5, and C2 is the center of the axis for nodding motions.

The manual 3D‐3D registration of the kVCBCT to the simulation CT was based on visual inspection of superimposed transverse, coronal and sagittal slices reconstructed from the two scans. Again, overall agreement of the cervical vertebra – especially C2 – positions was important for the registrations. Additional translational shifts to compensate apparent setup errors of above 1 mm were applied. Following the shifts based on the 3D‐3D registration, another manual 2D‐2D registration of a second pair of kV radiographs to DRRs was performed, and couch shifts were applied if the setup errors were judged to be above 1 mm.

The image‐guided setup in this study is defined as the entire series of couch corrections from each imaging step described above.

Immediately after treatment, another kVCBCT was acquired at treatment position to retrospectively evaluate intrafractional motion. A total of 47 post‐treatment kVCBCT were acquired.

### B. Data analysis

The coordinates of the setup errors are patient's left‐right (X), anterior‐posterior (Y) superior‐inferior (Z). The signs of rotations are right‐handed about each axis.

To independently assess the rotation errors at treatment positions, an automatic 3D rigid‐body registration of each pretreatment kVCBCT to the corresponding simulation CT was retrospectively performed on ROI's of two or three cervical vertebrae including C 2 using in‐house software that accounted for translations and rotations (six degrees of freedom, DoF). The software uses an intensity‐based algorithm with mutual information as the similarity measure^(^ ^21^ ^)^ and downhill simplex as the optimization algorithm.^(^ ^22^ ^)^ The quality of the registration was evaluated by visual inspection of the ROI's on the superimposed images in transverse, coronal and sagittal slices. For this retrospective study, the isocenter of the pretreatment kVCBCT study was always placed at the final treatment isocenter.

Finally, intrafractional errors were calculated by registering each pair of pre‐ and posttreatment kVCBCTs using the automatic 3D rigid‐body registration in three and six DoF.

## III. RESULTS

### A. Setup errors

**Figure 1:**
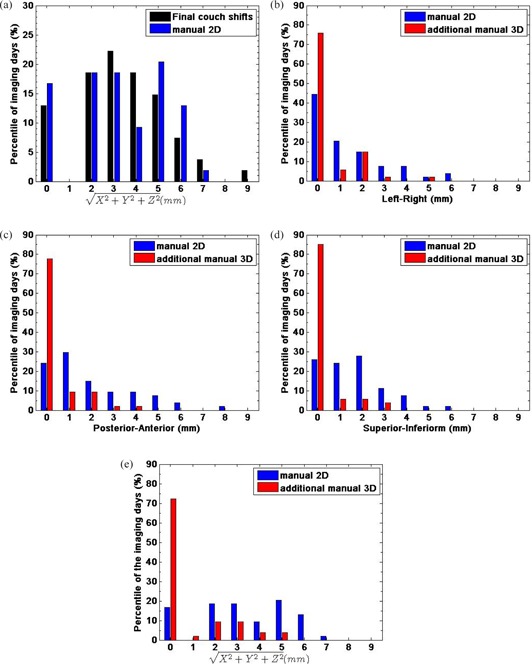
The percentile distributions of the 3D lengths of the final shifts: manual 2D‐2D followed by manual 3D‐3D (black) and the couch shifts determined by manual 2D‐2D (blue) are shown (a). The percentile distributions of the translational couch shifts determined by manual 2D‐2D registration (blue) from the initial patient position, and further shifts from the following manual 3D‐3D registration (red) for all 54 sessions in left‐right, X (b), posterior‐anterior, Y (c), superior‐inferior, Z (d) and the 3D length of the error vectors (e).

The final couch translations from the initial setup resulting from the series of manual 2D‐2D and 3D‐3D registrations at treatment ranged from 0 to 8 mm, and 85% of them were ≥2 mm in at least one direction. The average 3D length of the couch shifts over all the sessions was 3.5±2.0mm, and 67% of the sessions were ≥3 mm (black bars in Fig. 1(a).

For 81% of the imaging sessions, couch corrections from the initial setup position determined by the manual 2D‐2D registration were ≥2 mm in at least one direction, and ranged from 0 to 8 mm in any one direction (blue bars in Figs. 1(b), (c) and (d)).

The average absolute values of the couch corrections determined by the manual 2D‐2D registration over all 54 imaging sessions were 1.3±1.6, 2.0±1.9, 1.6±1.4 (± standard deviation, mm) in the X, Y and Z directions, and 3.5±2.2mm in the 3D length. The manual 3D‐3D registration following the manual 2D‐2D resulted in many fewer changes: 24% of the sessions had further shifts of ≥2 mm (red bars in Figs. 1(b), (c) and (d)). The absolute values of the further shifts determined by the manual 3D‐3D ranged from 0 to 5 mm and the averages over all imaging sessions were small: 0.5±1.0, 0.4±0.9, 0.3±0.7 and 0.8±1.5mm in the X, Y and Z directions and 3D length, respectively. Two patients had no couch shift from the manual 3D‐3D registration during the whole treatment course. The percentile distributions of the 3D lengths of the couch shifts determined by the manual 2D‐2D (blue bars in Fig. 1(e) show that for 67% of the sessions, the shifts were ≥3 mm, and no further shifts were made for 72% of the sessions from the addition of the manual 3D‐3D (red bars in Fig. 1(e). The couch shifts determined by the manual 2D‐2D registration were similar to the final couch shifts determined from series of the manual 2D‐2D and 3D‐3D registrations, as shown in the distributions of the 3D length of the setup error vectors (Fig. 1(a) where black bars are for the final couch shifts and blue bars for the manual 2D‐2D registration).

**Figure 2:**
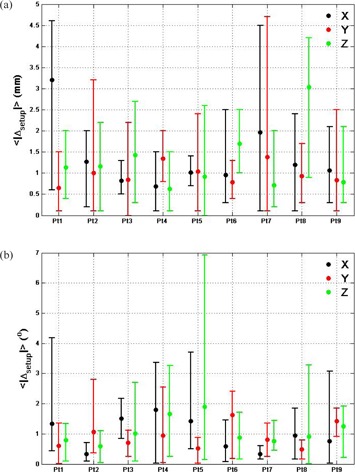
Average translational (a) and rotational (b) setup errors calculated through automatic six DoF registration at treatment position in X (black), Y (red) and Z (green) for all 54 imaging sessions. The bars are the range of the errors.

Each patient's average estimated translational and rotational setup errors in each direction at treatment positions as calculated with the automatic six DoF registrations are presented in Fig. 2(a) and Fig. 2(b), respectively; the averages are shown as points and the ranges as error bars. The average translational errors in each direction were < 2 mm except for the X and Z directions for patient #1 and #8, where average translational errors were 3.2 and 3.0 mm, respectively. The maximum of the translational errors was < 5 mm. The average rotational errors in each direction were small, averaging <2° with a range of 4°, except for patient #5 whose rotational error was 7° on one imaging day.

**Figure 3:**
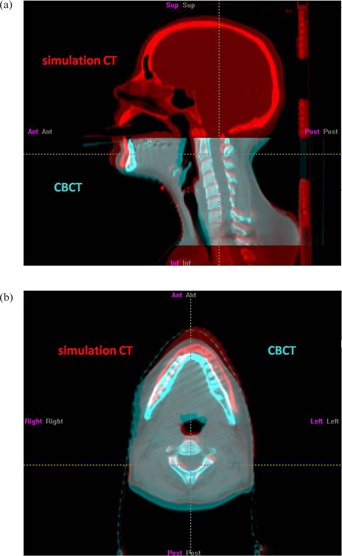
Sagittal (a) and transverse (b) views of patient #1 at treatment position (CBCT, cyan) on one imaging day superimposed with the simulation position (simulation CT, red). The patient's chin was dropped from the simulation position, and patient's anatomy was deformed. Note that the FOV of CBCT covered only part of the whole target volume for this patient.

Visual inspection showed obvious deformation of patient #1 and disagreement when automatic rigid‐body registration was performed on different ROIs (Fig. 3 and Table 2). The pre‐treatment kVCBCT at the treatment isocenter superimposed with the simulation CT (Fig. 3) shows that even when the C2 vertebra was well registered, the chin position was different from the simulation position. Table 2 shows the differences in the translational and rotational errors calculated on the ROI for manual registration (C2 & C3 vertebrae) and on the ROI of the mandible where the primary target was. The differences were large for all six imaging sessions for this patient.

**Table 2:** Differences between calculated translational and rotational errors on the ROI with C2 & C3 and the ROI with mandible for patient #1 on each imaging day. Translations are the lengths of the vectorial differences between two error vectors (C2 & C3 – mandible) and rotations are individual component of the difference vectors.

*Imaging Session*	*Differences of the Registration Results on the C2 & C3 and Mandible*			
	*Translation*	*X*	*Rotation (°) Y*	*Z*
1	5	−0.2	−0.9	1.7
2	8	4.7	−1.9	−0.2
3	5	1.0	−0.6	1.3
4	6	2.3	−0.1	0.3
5	10	1.3	1.1	1.5
6	4	−0.3	−0.6	1.1

### B. Intrafractional errors

**Figure 4:**
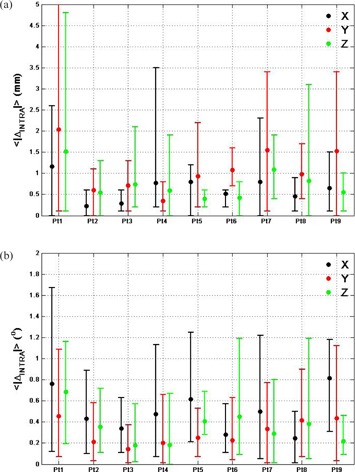
Average translational (a) and rotational (b) intrafractional errors calculated with automatic six DoF registration in X (black), Y (red) and Z (green) for all 47 post‐treatment kVCBCT. The bars are the range of the errors.

The average translational intrafractional errors determined by six DoF registration for each patient were ≤2 mm in any one direction (Fig. 4(a)). However, for patient #1 the intrafraction error was > 6 mm in the Y direction at one imaging session, which indicated substantial motion during treatment. The average rotational intrafractional errors were <1° for all patients, and rotation errors were always <2° in any axis (Fig. 4(b)).

## IV. DISCUSSION

We evaluated the improvement of setup errors using 2D and 3D image guidance for nine HNC patients initially positioned by aligning skin tattoos and lines on the customized thermoplastic mask with room laser. The image‐guided setup is a sequence consisting of manual 2D‐2D registration of orthogonal radiographs with DRRs and manual 3D‐3D registration of kVCBCT to the simulation CT, followed by a second manual 2D‐2D registration of another pair of kV radiographs. The results of this study are consistent with previous studies.^(^ ^2^ ^,^ ^23^ ^–^ ^24^ ^)^ For 63% of the imaging sessions, couch shifts determined by the first manual 2D‐2D registration were > 3 mm. When a small rotation was suspected based on the manual 3D‐3D registration, it was corrected by approximate simple translational shifts. For only 10% of the imaging sessions, further patient position corrections > 3 mm determined by the addition of manual 3D‐3D registration were applied. Our study indicates that 2D images, which are faster and easier to acquire and register than 3D images, substantially improve setup errors of a simple laser and tattoo alignment. However, registration with six DoF of the kVCBCT with the planning scan is useful in revealing rotational errors (which are too large to approximately compensate with translations) and also in revealing patient deformation. If a large rotation is observed in kVCBCT, the patient can be repositioned by removing the mask and putting it back, and then repeating the initial setup. However, without a six DoF couch or similar equipment, it is difficult to correct small rotations for the current HNC patient setup which involves a thermoplastic mask and head cushion. Adding 3D imaging without accounting for rotation in both registration and correction does not yield large benefits over 2D imaging. Also, kVCBCT has its limitation in imaging HNC patients. The contrast of kVCBCT is not good enough for direct registration on a soft tissue target and, as a result, registration can be performed only on bony anatomy. The field of view (FOV) of the Varian OBI kVCBCT is too small to image the whole target volume for large‐volume diseases such as nasopharyngeal cancer, which prevents accurate dose calculation based on the kVCBCT.

The rigid‐body registration results for patient #1, whose primary target volume was in the oral cavity and whose mask was loose, strongly depended on the chosen ROI. For this case, a better ROI for manual registration would be the mandible. The automatic 3D‐3D registration results on the ROI tightly defined around C2 & C3, and ROI similarly defined around the mandible were very different because the patient was deformed (Fig. 3 and Table 2).

To study dosimetric effects of patient positioning corrections via image‐guided setup, we estimated the dose to the high‐dose PTV for patient #9 whose corrections at treatment were the largest (up to 6 mm in one direction), except for patient #1. First, we transformed the PTV on the simulation CT to the treatment position by applying the inverse of the six DoF transformation matrix determined via automatic 3D‐3D registration of the kVCBCT to the simulation CT. Then we further moved the PTV to the initial (laser and tattoo alignment) setup position from the treatment position. We separately recalculated the doses on the PTV transformed to the treatment position and to the initial position, and compared D95 (dose encompassing the hottest 95% of the volume) and V95 (volume receiving >95% of the prescribed dose). On one imaging day, the reduction in D95 and V95 at the treatment position compared to the initial setup position were 1%, but on another day they were 1% higher at the initial position than at the treatment position. Our dose calculation indicated that the change in dose for the PTV was 1%~2% and the image‐guided setup did not always improve the dose coverage on a target. However, many HNC patients' targets are distributed over a large region including the whole neck, and therefore a deformable registration is needed to accurately calculate the delivered dose. The need to replan treatment or to remake the mask for resimulation could then be considered if the calculated delivered dose is significantly different from the original plan.^(^ ^25^ ^–^ ^28^ ^)^

Intrafractional errors showed that all patients stayed at the treatment position < 3 mm during treatment, except for patient #1 with poorly fitted mask.

Our study indicates that carefully made thermoplastic mask is critical to obtaining reproducible patient positioning and intrafractional immobilization.

## V. CONCLUSIONS

Manual 2D‐2D registration on orthogonal‐pair images resulted in translational correction up to 8 mm relative to the laser/skin tattoo setup. Our results show that 2D imaging is sufficient to reduce setup errors on most imaging days. 3D‐3D registration of the planning scan with a pretreatment kVCBCT without accounting for both translational and rotational errors in registration and correction is only rarely beneficial over manual 2D‐2D registration. Our study indicates that patient position corrections determined by manual 2D‐2D are greater than 5 mm for 30% of imaging days, and daily 2D imaging is useful to find these outliers. Based on these observations, we suggest that daily kV 2D‐2D registration on the ROI of in‐field bony anatomy is the optimal setup method for HNC patient in terms of balance between positioning improvement and easy implementation at treatment. However, kVCBCT is useful for detecting patient rotations, which cannot be corrected by simple translations, and patient deformation large enough to require a repeat of the setup or even – if problems persist – construction of new immobilization and resimulation. Our study also shows that a well‐fitting thermoplastic mask is critical for effective positioning and immobilization; therefore, special attention is needed while mask is hardening at simulation.

## ACKNOWLEDGEMENTS

This work was supported by a NIH training award, T32‐CA61801.
